# Mental Dynamics, or Groundwork of a Professional Education

**Published:** 1847-07-01

**Authors:** 


MENTAL DYNAMICS, OR GROUNDWORK OF A PROFESSIONAL EDUCATION. THE HUNTERIAN ORATION BEFORE THE ROYAL COLLEGE OF SURGEONS OF ENGLAND, 15th FEBRUARY, 1847. By *Joseph Henry Green*, F.R.S. late Professor of Anatomy and Surgery to the College: Professor of Anatomy to the Royal Academy: one of the Surgeons to St. Thomas's Hospital. Octavo, pp. 65. Pickering, London, 1847.

No one present in the Theatre of the College of Surgeons on the 15th of February last will soon forget the discourse to which they listened on that day. For one hour and three quarters Mr. Green commanded the earnest attention of a densely crowded audience, by a display of learning and eloquence, in an address delivered with consummate skill and brilliant effect. But on calmly reading this oration in the closet, we could not but be impressed, how greatly its success was due to the happy and effective delivery of the orator. It contains, indeed, metaphors and passages of great beauty, but at the same time much that is impracticable, unintelligible, and in the obscure style of Coleridge.

Mr. Green, after paying a brief but graceful tribute to the distinguished services of John Hunter, states that his present purpose is not that of dwelling on his merit as a physiologist and surgeon; but of considering how far his excellence may be deemed a pattern for the formation of a scientific character in unison with the requirements of our profession.

> "We are here indeed, on the very threshold of our enquiry, met by a difficulty, which can neither be overlooked nor disregarded; it is this, that the predominant and characteristic trait of Hunter's mind was Genius. Now if, in accordance with almost universal belief, we admit without qualification that this attribute implies a perfection unattainable by human effort—that it is absolutely a special gift of Providence imparting to the mind of its possessor somewhat of the power originative and creative of the Divine Giver—we must humbly confess that Genius is not an imitable acquirement, and we shall be constrained to abandon as hopeless any search for the means of emulating Hunter's peculiar excellence by mental culture and self-exertion. Nevertheless, who will deny, that by educing and cultivating the powers, which any fairly endowed individual may possess, we may preserve the freshness, improve the vigour, and favor the originative faculties of the mind? And if, as cannot be doubted, the art and science of healing eminently require a mind relying on its own resources, it cannot be unworthy of our study how the mind may be best trained in order to elicit its inherent powers and native energies, and to render that calculable and regular, which otherwise would be but a happy accident." P. 8.

Hunter unquestionably possessed genius in its truest and highest sense, but in that sense we can scarcely regard this attribute as an "imitable acquirement;" nor should we exactly select him as a pattern for the rising generation except for his "unceasing application and marvellous industry," without which, as Mr. Green states, "we should find original power elanquesce, and exhaust itself in immature and abortive productions." Much as Hunter has accomplished, we can come to no other conclusion than that this self-taught genius might have achieved still more, and at least have done fuller justice to the productions of his own intellect, had his powers been cultivated and his mind disciplined in early life; in fact, had he enjoyed the advantages of the training so forcibly inculcated in this discourse.

The object of the Oration is an attempt to define the intellectual discipline "which may best prepare the mind for the scientific cultivation of a profession, and aid the individual in forming his character in the light of a final aim." We cannot follow Mr. Green in the learned arguments by which he shows the value of a knowledge of letters, natural history, the history of man, mathematics, logic, &c. We must be content with the selection of a few passages which will enable our readers to form some notion of the style of the author, and of the elevated character of his sentiments.

Mr. Green describes Natural History as—

"A study, which offers food for every digestion, knowledge for every capacity, and which tends more perhaps than any other to people, to enlarge, and to tranquillize the mind. But where begin, or where end, in the vast living panorama of God's works, in which earth, air, sky and ocean, teeming with the multitudinous assemblage of their countless myriads of products and inhabitants, form the mighty canvass, and rise upon the overwhelmed sense of the youthful student, as they may have burst upon the view of our first progenitor, and still as fresh as in the morning of creation? The 'majestical roof of heaven fretted with golden fire;'—ocean and the watery world, as the workshop where the busy artist Nature prepares the first rude sketches of the living forms, which she afterwards brings to view in a higher and more perfected character;—mid-air, with its shifting scenery of light and clouds, its meteors and changeful variety of atmospheric phœnomena;—and earth, with its hidden treasures of metal, chrystal, and precious gem; its surface, diversified by river, lake and mountain, adorned with all the forms of living growth from the lofty forest tree to the lowly and lovely flower, and peopled by the animated tribes, that form the graduated scale of organic life. Here Curiosity is ever excited, Attention rivetted, and Memory bribed, by perpetual novelty, variety and beauty;—the Comparing power is ever kept alive by an endless succession of similitudes and contrasts, that now sustain the interest by inducing the pupil to note the like in the different and the different in the like, and now re-awaken the flagging attention by renewed excitement and gratification of the senses;—and the Reasoning Power is finally evoked in order to trace and explain the varying adaptation of means to proximate ends, displayed in Instincts which anticipatively rehearse the functions of that faculty, which when enlightened by Reason, and directed to ultimate ends, becomes Human Understanding. Thus, as the student watches the ascension of nature into mind, he shall learn that, up the whole ascent, nature is a prophetic-hymn, heralding the advent of man, and proclaiming the wisdom and goodness of the Creator." P. 19.

We may quote the following remarks on a subject much neglected in the education of medical men, viz. Logic. Its proper province is

"Reasoning or Discourse—the process by which we deduce from known truths all that they legitimately comprehend, by which we apply general rules to particular cases, by which we infer from some less comprehensive truth one of more comprehensive generality;—it is the process by which we weigh evidence, infer and prove by argument, and draw universal and necessary conclusions;—while in every judgment the presence of Reason is attested by the claim, which it asserts and vindicates to the unavoidable conviction of all rational beings.

And the results are the Rules, Maxims and Judgments, which constitute our generalized Experience.

It is not therefore the art of one

> ' Profoundly skill'd in analytic ;
> Who can distinguish and divide
> A hair 'twixt south and south-west side ;
> On either which who can dispute,
> Confute, change hands, and still confute :'—
>
> BUTLER.

" Reasoning is the daily and hourly business of our lives, and, whatever our worldly calling—in the pulpit, at the bar, or at the bedside of the patient—we are unceasingly occupied in inferring or proving a something from what is already ascertained or taken for granted. And in the words of an eminent writer on Logic, ' To learn to do that well, which every one will and must do, whether well or ill, may surely be considered as an essential part of a liberal education.' " P. 31.

We shall conclude this notice with an extract expressive of Mr. Green's approval of the institution of collegiate establishments at some of the principal medical schools.

" I have only to express my unaltered conviction, strengthened indeed by the success with which the trial has been already attended,—that it is in Universities and Colleges that a medical education may be best grounded on those universal elements of science, which are the essential constituents of every liberal profession. I hold it indeed scarcely possible that any professional education can be fully accomplished except in such Institutions ;—where discipline both intellectual and moral, and a pledged direction and supervision of the studies, give the requisite security for its progress and completion ;—and where the *Alumni* are induced habitually to regard themselves as members of one body, and to form among themselves a correspondent law of honor, of self-respect, and of respect for each other as fellow-collegians—with the cognate habit of despising the hollow, the tricky, and the ostentatious,—in short, to form that sentiment of honor and gentlemanly feeling, in which the moral life of the individual breathes as in its natural atmosphere, with an unconsciousness, which gives the charm of unaffected manners and conduct." P. 42.

